# Renal Cell Carcinoma Mimicking Adrenal Tumor

**DOI:** 10.1155/2010/987128

**Published:** 2010-08-23

**Authors:** Mohammad Kazem Moslemi, Shabir Al-Mousawi, Mohammad Hasan Dehghani Firoozabadi

**Affiliations:** ^1^Department of Urology, Kamkar Hospital, School of Medicine, Qom Medical Sciences University, 3715694978 Qom, Iran; ^2^Division of Urology, Department of Surgery, Al-Amiri Hospital, 13041 Kuwait City, Kuwait; ^3^Department of Urology, Shahid Rahnemoon Hospital, School of Medicine, Yazd Medical Sciences University, Yazd, Iran

## Abstract

There are a variety of causes of adrenal pseudotumors on computerized tomography (CT) scan, including upper-pole renal mass, gastric diverticulum, prominent splenic lobulation, pancreatic mass, hepatic mass, and periadrenal varices. We present a case of a large subhepatic mass that discrimination of its origin from neighborhood organs was difficult preoperatively. Our patient was a 58 years old man, that three months after an unsuccessful operation in another center for a pseudoadrenal mass underwent a very difficult subcapsular tumorectomy in our center.

## 1. Case Presentation

**Figure 1:**
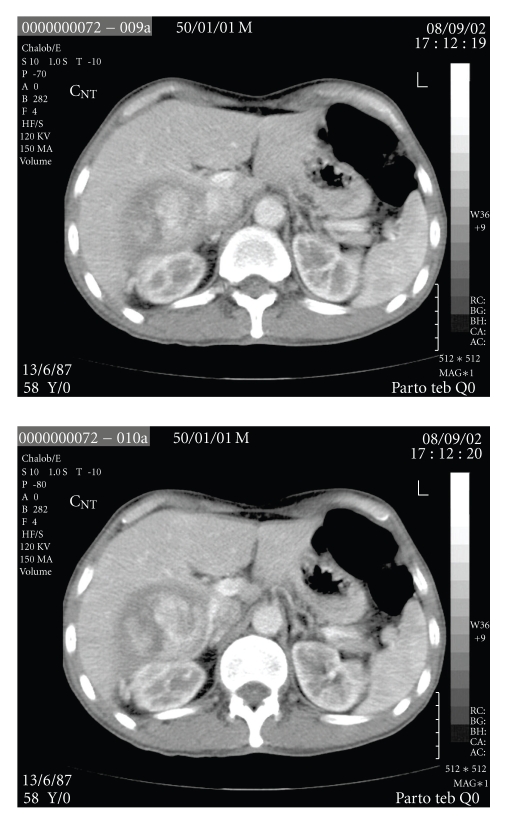
CT scan of the patient revealing the subhepatic mass.

**Figure 2:**
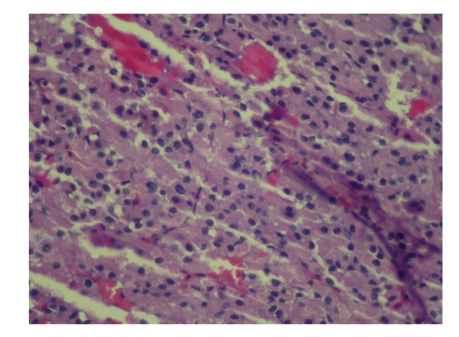
The histopathological examination (x100 HPF) of the tumorectomy with H&E revealed clear cells of RCC.

The patient is a 58 years old Iraqi male that referred to our center three months after an unsuccessful operation for removing a subhepatic mass, probably adrenal mass. He experienced general weakness, fatigue, and weight loss in the last several months. The patient BMI was 20 kg/m^2^. In physical examination, vital signs were normal. In abdominal examination, a scar of previous surgery in the right subcostal area (*subcostal incision*) with near 15 cm length was noted. In hematological examination, Hb was 11 g/dl. In serum biochemistry, renal function tests, electrolytes, and bilirubin were all within normal limits. Only mild elevations of hepatic enzymes were noted. Serum and urinary catecholamines were normal. Serum levels of cortisole and aldosterone were normal. In CT scan that was done with intravenous and oral contrast (Figure 1), a large mass located in the subhepatic area and intervening with the upper pole of the right kidney was noted. In the report of previous operation that was done in Iraq, tumorectomy was failed due to severe adhesions of the mass to the peripheral tissues. This mass was isolated from the liver. The patient scheduled for right adrenalectomy with the midline laparotomy incision. After opening the layers, the mass was located in the lodge of upper pole of the right kidney. It was severely adherent to the peripheral tissues, due to severe adhesion of the mass to the subhepatic area, its differentiation from the kidney or adrenal was difficult. Due to severe bleeding from the subhepatic bed subcapsular resection of the mass with the adherent kidney performed. Its total size was 14 cm × 10 cm and it weighted 2250 grams. The final Histopathological examination of the mass revealed, renal cell carcinoma, clear cell type, Fuhrman nuclear grading 2 (Figure 2) with invasion to Gerotas fascia and involvement of ipsilateral adrenal which originated from the upper pole of the kidney as exophytic growth pattern (T3N0M0). The postoperative course was uneventful and the patient discharged home at fourth postoperative day uneventfully. The patient was well one year after operation without any chemo or radiotherapy.

## 2. Discussion

The adrenal glands are surrounded by a variety of anatomical structures. Certain anatomical structures and extra-adrenal pathological conditions may produce CT images suggesting adrenal pathology where none actually exists. Disorders of the adrenal gland result in classic endocrine syndromes such as Cushing syndrome, hyperaldosteronism, and pheochromocytoma. In addition, tumors of the adrenals may present with abdominal pain or as an abdominal mass. The diagnosis of these disorders requires careful endocrine evaluation, and in many patients adrenal imaging studies are required to define adrenal anatomy. In our patient, pulse, blood pressure, renal function tests, serum cortisole, electrolytes, and urinary catecholamines were all normal. Preliminary diagnosis was therefore nonfunctioning adrenal tumor. However, the tumor located below the right adrenal gland during operation and the diagnosis of renal cell carcinoma (RCC) depended on the histopathological examination. A mass arising in the upper pole of the kidney may be difficult to differentiate from an adrenal mass [1, 2]. The reported incidence of adrenal incidentaloma identified in conventional radiographic examinations is approximately 5% [3]. Additional examinations are usually necessary to confirm the nature of the lesion and to exclude the presence of life-threatening malignant disease. Intraoperative pathology consultation for kidney specimens is requested infrequently. In one study, only 21 of 1000 specimens submitted for frozen section diagnosis were of genitourinary origin [4], which reflects the relatively low incidence of kidney lesions that necessitate surgery and the limited role of frozen section in the surgical management of these lesions. With the advancements in the diagnosis and treatment of renal tumors, frozen section has become more important. Frozen section usually is requested for synchronous renal and adrenal tumors. Differentiating an adrenal adenoma from a metastatic clear cell RCC can be difficult [5]. F-fluro-2-deoxy-D-glucose positron emission tomography (FDG-PET) has proved to be valuable in the diagnosis and management of a variety of malignancies, but is still limited in providing detailed anatomical information. According to the literature, an adrenal incidentaloma with high FDG uptake usually indicates malignancy and requires further investigation. However, accurate localization of the adrenal gland in FDG-PET is difficult without the presence of surrounding well-visualized organs, such as the kidney or liver. If these organs have a congenital anomaly or are altered due to a previous operation, misdiagnosis can occur [6]. A rare diagnosis when faced with a renal mass is that of an ectopic adrenal adenoma. Distinguishing this entity from renal cell carcinoma has proven to be extremely difficult due to similarities in clinical presentation and radiologic findings. Typically, the diagnosis of an ectopic adrenal adenoma is only reached after surgical excision and careful pathologic examination [7]. Frozen section usually is requested for synchronous renal and adrenal tumors. Differentiating an adrenal adenoma from a metastatic clear cell RCC can be difficult. An ipsilateral adrenal mass in a patient with RCC is not uncommon and usually is a primary adrenal lesion rather than metastatic RCC [5]. On the basis of Takeda et al. [6] study, I-131 cholesterol adrenocortical scintigrapgy is useful in the differentiation of adrenal and renal tumors when a large tumor occupies the upper pole of the kidney or adrenal gland. In FDG-PET, a normal right adrenal gland is barely visualized [8], whereas an adrenal lesion with intense FDG uptake usually represents malignancy [8–10]. Finally, possible causes of the adrenal pseudotumors [11] are as follows; Exophytic upper pole renal mass, abundant suprarenal fat, retroperitoneal tumor localized in the area of adrenal gland, prominent lobation of the hepatic lobe, or hepatic tumor, gastric diverticulum and redundant gastric fundus, fluid filled duodenum/colon, splenic lobulation, tortuous or dilated splenic arteries and veins, pancreatic tail in an unusual location or pancreatic tail mass, and focal thickening of adjacent diaphragmatic crus.

## 3. Conclusion

Although some of the imaging modalities like meticulous CT technique, MRI (with emphasis on the usefulness of three-dimensional reconstructions) or Intraoperative frozen section can help in the differentiation between large masses of adrenal and/or renal tumors, but the final diagnosis depends on the histopathological examination by skilled pathologists. In these instances, diagnostic pitfalls did not affect patient care, because these cases generally are candidates for adrenalectomy or radical nephrectomy.
